# RACIAL AND ETHNIC DIFFERENCES IN PARTICIPANT EXPERIENCE OF HOME- AND COMMUNITY-BASED SERVICES

**DOI:** 10.1093/geroni/igac059.198

**Published:** 2022-12-20

**Authors:** Howard Degenholtz

**Affiliations:** University of Pittsburgh, Pittsburgh, Pennsylvania, United States

## Abstract

It is widely recognized that racial and ethnic minorities experience lower quality of care and worse health outcomes across a wide range of health services and long-term care settings. However, less is known about racial and ethnic differences in home and community-based services (HCBS). This study used the HCBS version of the Consumer Assessment of Health Providers Survey (CAHPS) to examine participant experience of care in a large, statewide Medicaid program. The CAHPS-HCBS is a validated measure of quality of HCBS that supplements measures of health outcomes and provides policy makers with critical data on system performance. As part of a larger evaluation of Pennsylvania’s transition to Managed care for users of HCBS, representative samples of older adults were interviewed before and after the program was implemented. This study takes advantage of the phased implementation of managed care to estimate the causal effect of the program on differences in participant experience by racial and ethnic group. In general, non-Hispanic whites rated their overall satisfaction with HCBS lower than non-Hispanic Black and Hispanic people. The implementation of Managed care was associated with improvements in non-Hispanic Black and Hispanic ratings of personal care and service coordination, but not that of non-Hispanic whites. One implication of these findings is that managed care organizations can have positive impact on participant experience. The CAHPS-HCBS instrument is publicly available and can be used by state agencies to monitor racial and ethnic disparities in participant experience.

